# Asymptomatic bacteriuria in critical-access hospitals: Prevalence and patient characteristics driving treatment

**DOI:** 10.1017/ice.2023.220

**Published:** 2024-03

**Authors:** Whitney Hartlage, Chloe Bryson-Cahn, Alyssa Y. Castillo, Rupali Jain, John B. Lynch, Natalia Martinez-Paz, Jeannie D. Chan, Zahra Kassamali Escobar

**Affiliations:** 1 School of Pharmacy, University of Washington, Seattle, Washington; 2 Center for Stewardship in Medicine, University of Washington, Seattle, Washington; 3 Division of Allergy and Infectious Diseases, University of Washington School of Medicine, Seattle, Washington; 4 Division of Infectious Diseases, University of Colorado, Aurora, Colorado; 5 Fred Hutchinson Cancer Center, Seattle, Washington

## Abstract

We evaluated the prevalence and treatment of asymptomatic bacteriuria (ASB) in 17 critical-access hospitals. Among 891 patients with urine cultures from September 2021 to June 2022, 170 (35%) had ASB. Also, 76% of patients with ASB received antibiotics for a median duration of 7 days, demonstrating opportunities for antimicrobial stewardship.

“Critical access hospital” (CAH) is a Center for Medicare & Medicaid Services (CMS) designation given to rural hospitals with <25 inpatient beds and located >35 miles from another hospital. Reports suggest that antimicrobial stewardship practices should be tailored to the local facility environment; however, published data to guide stewardship interventions are limited in rural and CAHs.^1^

Appropriate testing for urinary tract infections (UTIs) and avoiding treatment of asymptomatic bacteriuria (ASB) are important antimicrobial stewardship targets.^2^ A recent analysis including 84 patients noted both high prevalence and overtreatment of ASB in CAHs. With >1,350 CAHs in the United States, improved awareness of testing and treatment related to ASB on a larger scale would be useful to inform implementation of stewardship initiatives in these settings. We evaluated the prevalence of ASB and proportion of treated ASB in a cohort of CAHs.

## Methods

This multisite, quality improvement study included 17 CAHs participating in the University of Washington Center for Stewardship in Medicine (UW CSiM), formerly known as the University of Washington Tele-Antimicrobial Stewardship Program (UW-TASP). UW CSiM is a collaborative between the University of Washington and 87 community hospitals, rural hospitals, and CAHs. In this study, we focused on implementing stewardship in CAHs by targeting the inappropriate diagnosis and treatment of ASB. All sites received prior education about UTIs through their participation in UW-CSiM, and 1 of 17 CAHs implemented an intervention to eliminate automatic urine analyses in long-term care patients, 2 months prior joining the quality improvement cohort. All sites participated in the UW CSiM intensive quality improvement cohort, through which hospitals received didactic education, individual coaching, peer mentorship, and support for data collection and analysis.

Abstractors at each CAH retrospectively identified and collected data between September 1, 2021, and June 10, 2022, using a REDCap electronic data collection tool.^4^ Patients aged ≥18 years with a urine culture collected during an ambulatory or inpatient healthcare encounter were included. A positive urine culture was defined as 1 or more species of bacteria growing in the urine at ≥100,000 colony-forming units (CFU)/mL. ASB was defined as a positive urine culture without any documented signs or symptoms of UTI, according to the National Hospital Safety Network (NHSN) definition and Infectious Diseases Society of America (IDSA) guidelines: temperature > 38.0°C, suprapubic tenderness, costovertebral angle pain or tenderness, urinary urgency or frequency, dysuria, hematuria, and altered mental status plus a systemic sign of possible infection (peripheral leukocytosis >10,000 cells/mm^3^, systolic blood pressure <90 mmHg, or ≥2 criteria for systemic inflammatory response syndrome [SIRS]).^2,5^ Patients who were pregnant, who were receiving antimicrobials for a concomitant bacterial infection, or for whom relevant details were missing during data collection were excluded.

The primary outcomes were the prevalence of ASB and proportion of ASB cases that received treatment, defined as the percentage of individuals with ASB who were prescribed an antibiotic. No statistical testing was performed. The study was reviewed by the University of Washington institutional review board, and approval was not required.

## Results

We reviewed the data for 1,087 patients with urine cultures; the median number of cases submitted per CAH was 45 (IQR, 34–90). Among the patients identified, 891 (82%) were included. Exclusions were due to treatment for concomitant bacterial infections (n = 106), missing data (n = 65), age <18 years (n = 17), and pregnancy (n = 8). Overall, 75% were female, and the median age was 69 years. The emergency department (ED) was the most common location for urine culture collection (72%). Also, 75% of urine cultures originated from positive urinalysis results reflexing to culture. Baseline characteristics are summarized in Table 1.

**Table 1.** Study Population Baseline and Clinical Characteristics

Variable		Asymptomatic Bacteriuria, No. (%)^a^		
	All Cases (n=891)	All (n=170)	Treated (n=129)	Not Treated (n=41)
Sex, female	667 (75)	132 (78)	95 (74)	37 (90)
Age, median y (IQR)	65 (41–78)	75 (64–85)	78 (65–85)	70 (64–83)
Urological comorbidities^b^	147 (17)	28 (17)	25 (19)	3 (7)
**Location of urine culture collection**				
ED, then admitted	190 (21)	45 (27)	34 (26)	11 (27)
ED, then discharged	448 (50)	82 (48)	64 (50)	18 (44)
Ambulatory care clinic	151 (17)	33 (19)	23 (18)	10 (24)
Other outpatient^c^	87 (10)	7 (4)	6 (5)	1 (2)
Inpatient	14 (2)	3 (2)	2 (2)	1 (2)
Documented signs and symptoms of urinary tract infection	560 (63)	…	…	…
≥2 SIRS criteria^d^	121 (14)	20 (12)	8 (6)	4 (10)
Positive blood culture within 72 h of urine culture	17 (2)	…	…	…
Altered mental status alone	32 (4)	15 (9)	15 (12)	0 (0)
Urinalysis that reflexed to culture	667 (75)	160 (94)	123 (95)	37 (90)
**Positive urinalysis result**				
Leukocyte esterase	626 (70)	131 (77)	108 (84)	23 (56)
WBC >10 ×10^9^/L	449 (50)	89 (52)	75 (58)	14 (34)
WBC <10 ×10^9^/L	244 (27)	48 (28)	33 (26)	15 (37)
Nitrites	309 (35)	82 (48)	68 (53)	14 (34)
**Bacteria type among those with positive urine cultures**				
Positive urine cultures	486 (55)	170 (100)	129 (100)	41 (100)
*Escherichia coli*	336 (69)	110 (65)	86 (67)	24 (59)
*Klebsiella* spp	52 (11)	16 (9)	13 (10)	3 (7)
*Enterococcus* spp	32 (7)	11 (6)	8 (6)	3 (7)
*Proteus mirabilis*	29 (6)	7 (4)	5 (4)	2 (5)
*Pseudomonas aeruginosa*	19 (4)	7 (4)	6 (5)	1 (2)
*Citrobacter* spp	11 (2)	2 (1)	1 (1)	1 (2)
*Enterobacter* spp	11 (2)	3 (2)	3 (2)	0 (0)
Presence of MDR bacteria^e^	21 (4)	8 (5)	8 (6)	0 (0)

Note. IQR, interquartile range; ED, emergency department; SIRS, systemic inflammatory response syndrome; WBC, white blood cell count; MDR, multidrug-resistant.

a Units unless otherwise specified.

b Urological comorbidities included chronic indwelling urinary catheter use, chronic intermittent straight catheterization, urologic procedure in last 30 d, urinary retention, neurogenic bladder, abnormal urinary anatomy (eg, nephrostomy, urinary stent, ileal conduit; and did not include horseshoe or solitary kidney).

c Other outpatient includes rehab or long-term care, urgent or quick care facility, or home health.

d Meeting sepsis criteria includes ≥2 criteria for systemic inflammatory response syndrome [SIRS]; temperature >38°C or <36°C, heart rate >90 beats per minute, respiration rate >20 breaths per minute, WBC >10 ×10^9^/L.

e MDR bacteria included the presence of extended-spectrum β-lactamase (ESBL)–producing bacteria, vancomycin-resistant *Enterococcus* (VRE), or carbapenem-resistant Enterobacterales (CRE).

Among 486 patients with a positive urine culture, 170 (35%) had ASB, and 129 (76%) received antibiotics. We detected a higher proportion of older age, male, urological comorbidities, and acute mental status changes among those treated for ASB. Among the 129 patients with ASB treated with antibiotics, oral agents were prescribed for 105 (81%). Moreover, β-lactams (45%) were most frequently prescribed, followed by nitrofurantoin (19%) and fluoroquinolones (18%) (Table 2). Of the 55 patients who received intravenous therapy, 98% received a β-lactam. The median antibiotic duration was 7 days (IQR, 3–7). Also, 95% of patients treated for ASB had a positive urinalysis that resulted in a reflex to culture.

**Table 2.** Antibiotic Prescribing among Patients without Urinary Symptoms

Variable	Treated ASB (N=129), No. (%)^a^
Received any oral therapy^b^	105 (81)
β-lactam^c^	47 (45)
Fluoroquinolone^d^	19 (18)
Nitrofurantoin	20 (19)
Trimethoprim-sulfamethoxazole	12 (11)
Received intravenous therapy during hospitalization	55 (43)
β-lactam^e^	54 (98)
Fluoroquinolone^d^	1 (2)
Received intravenous therapy at discharge	1 (0.8)
Duration of therapy, d (IQR)	7.0 (3–7)

Note. IQR, interquartile range.

a Units unless otherwise specified.

b Received any oral therapy includes during hospitalization, at discharge, or in the outpatient setting.

c Oral β-lactam agents includes amoxicillin, amoxicillin-clavulanate, cefdinir, cefpodoxime, cephalexin.

d Fluoroquinolone agents include levofloxacin and ciprofloxacin.

e Intravenous β-lactam agents includes ampicillin-sulbactam, cefazolin, cefepime, ceftriaxone, ertapenem, meropenem, piperacillin-tazobactam.

Among 405 patients with a urine culture showing <100,000 CFU/mL of bacterial growth (including those with no growth), 160 (40%) had no documented signs or symptoms of UTI. More than half (59%) were treated with antibiotics for a median duration of 7 days (IQR, 3–7).

## Discussion

To our knowledge, this study is the largest to evaluate the prevalence and treatment of ASB in CAHs. Importantly, 35% of patients with a positive urine culture had no documented UTI-related symptoms.

Although the prevalence of ASB is notably lower in our study compared to previous reports (45%–71%), 75% of patients were inappropriately prescribed antibiotics for ASB.^3,6,7^ This finding is consistent with other studies that also reported a high rate of overtreatment.^3,6–8^ Although a growing body of evidence demonstrates the lack of clinical benefit with treatment of ASB, more concerning are the underrecognized data suggesting potential harm.^2^ Curren et al^9^ found that each day of antibiotic therapy was associated with 4% increased odds of experiencing an adverse drug event (ADE). Notably, 19% of ADEs have been attributed to antibiotic regimens that were not clinically indicated, most commonly because of treatment of ASB.^9^ In our study, the median duration of therapy was 7 days (IQR, 3–7) among treated ASB patients, which exceeded guideline recommendations for cystitis.^10^ Even for those patients with ASB who are inappropriately treated, the potential for antibiotic-associated harm could be reduced through decreased duration of therapy.^3,9,10^

In our study, most urine cultures was collected in the EDs of CAHs. Thus, EDs represent a high-yield location for stewardship interventions because urine culture is often ordered before initiating a symptom-driven workup as a triage to optimize ED throughput.^11^ In our study, almost half of the patients with ASB were discharged directly from the ED, which limits opportunities to re-evaluate appropriateness of therapy as new data become available, including urine culture results. Therefore, ED workflows are a particularly important target for ASB interventions in CAHs.

Interestingly, nearly 60% of asymptomatic patients received treatment despite urine cultures showing <100,000 CFU/mL of bacterial growth or no growth. We postulate that the strict definition of ASB undercaptures the overall inappropriate antibiotic prescribing.

Our study had several limitations. Given the retrospective nature of the study and lack of case selection standardization among sites, there was a potential for bias in the selection of patients across the spectrum of care in both ambulatory and hospital settings. Identification of abnormal urinalyses and criteria to reflex a urinalysis to culture varied from institution to institution. Duration of therapy was based on written prescriptions; therefore, we were not able to confirm whether the antibiotic course was completed or was subsequently discontinued. Lastly, there was an overrepresentation of cases from some sites and insufficient representation from others.

In summary, similar to the well-described overtreatment of ASB in larger, urban, academic hospitals, inappropriate treatment of ASB is common in CAHs, especially in their EDs. Unnecessary antibiotic use in patients with ASB and long duration of therapy in treated patients are important areas for stewardship interventions in the CAH setting.
